# Women empowerment and healthcare utilization for childhood illnesses: evidence from Ethiopia

**DOI:** 10.1093/inthealth/ihaf062

**Published:** 2025-05-31

**Authors:** Bazie Mekonnen, Abebe Gebremariam, Negussie Deyessa, John N Cranmer

**Affiliations:** College of Health Sciences, Addis Ababa University, Addis Ababa, Ethiopia; Woodruff Health Sciences Center, Emory University, Atlanta, GA, USA; College of Health Sciences, Bahir Dar University, Bahir Dar, Ethiopia; Emory-Ethiopia, Addis Ababa, Ethiopia; College of Health Sciences, Addis Ababa University, Addis Ababa, Ethiopia; College of Health Sciences, Bahir Dar University, Bahir Dar, Ethiopia; Emory-Ethiopia, Addis Ababa, Ethiopia; Center for the Study of Human Health, Emory University, Atlanta, GA, USA

**Keywords:** empowerment, healthcare seeking, under-five children, under-five illness, women

## Abstract

**Background:**

Five million children <5 y of age died globally in 2021, the majority (56%) in sub-Saharan Africa (SSA). Many of the deaths in children <5 y of age could be prevented through early detection and treatment. However, healthcare utilization for childhood illnesses remains low in the region. The aim of this study was to assess the relationship between women empowerment and healthcare utilization for childhood illnesses.

**Methods:**

The main predictor variable for this study was women’s empowerment and the outcome variable was healthcare utilization for childhood illnesses. The data source for the study was the 2016 Ethiopia Demographic and Health Survey dataset. Complex sample ordinal regression analysis was employed, controlling for confounders. Adjusted cumulative odds ratios (cuORs) and 95% confidence intervals were computed to estimate effect size.

**Results:**

A total of 2101 (weighted) cases of children <5 y of age were included in this study. Children whose mothers were empowered with a wife-beating attitude were 59% more likely to get healthcare for all illnesses in children <5 y of age (β=0.46, cuOR 1.59, p<0.01). Children whose mothers had no problems with healthcare access were also more likely to receive health services for all illnesses in children <5 y of age (β=0.42, cuOR 1.52, p=0.01).

**Conclusions:**

Women’s empowerment has a significant effect on healthcare utilization for illnesses in children <5 y of age. The finding indicates empowering women, sooner or later, is empowering the family.

## Introduction

Child mortality in sub-Saharan Africa (SSA) continues to be a persistent challenge.^1–3^ Five million children <5–y of age died worldwide in 2021 and 56% of the deaths occurred in SSA with an average mortality rate in children <5 y of age of 74 deaths per 1000 live births.^1^ Most childhood deaths occur from preventable causes. The infectious diseases pneumonia (15%), diarrhoea (8%) and malaria (5%) remain the leading causes of mortality globally in children <5 y of age.^1–3^

Many of the deaths from these illnesses in children <5 y of age could be prevented through early detection and treatment of cases. An ecological study to identify correlates of child mortality in 82 developing countries indicated that healthcare seeking for acute respiratory infections (ARIs) (correlation coefficient: −0.48), provision of oral rehydration salt (ORS) for diarrhoea (correlation coefficient: −0.52) and antimalarials given for children with fever (correlation coefficient: −0.56) had a significant protective effect on childhood mortality.^4^

However, the prevalence of healthcare seeking for illnesses in children <5 y of age remains low in high-burden countries such as in SSA.^1–3,5^ (Healthcare seeking and healthcare service utilization are used interchangeably for this study. Similarly, respondents, mother, women and caretaker are interchangeable.) In 2021, UNICEF’s estimate for the SSA region indicated 46% and 60% care-seeking prevalence for children with ARI symptoms and fever, respectively.^5^ The report showed that only 37% of children with diarrhoea received ORS.^5^ An ecological study in developing countries indicated that 49% of child caregivers sought care for childhood ARI symptoms from a health institution and 39% of children with diarrhoea received ORS.^4^ According to the study, only 13% of children with fever were given antimalarials.^4^ Studies in Nigeria and Kenya also indicated a care-seeking prevalence for childhood illnesses of 21.6% and 30.4%, respectively.^6,7^ These studies showed that maternal obstetric factors, service accessibility, service quality and cost were significant predictors of healthcare seeking for illnesses in children <5 y of age.

Research indicates that women’s empowerment has a significant effect on the health and general well-being of children. Empowerment is a process of change by which those who have been denied the ability to make strategic life choices—the powerless—regain their autonomy.^8–10^ It is a multidimensional concept grouped under three dimension pillars: resources (the financial, physical, human and social capital that enable choice); agency (the capacity to freely make decisions about one's own life) and self-efficacy (the belief in one's ability to act effectively towards a goal); and context (the social arrangements, mainly norms and institutions, necessary to express agency and assert control over resources).^8–10^ Empowerment involves removing barriers and creating an enabling environment for women to own and use these pillars.^8–10^

Women’s empowerment is a contextual concept as well—an empowerment indicator in one country/culture might not have a similar empowering effect in another. This feature of empowerment poses a challenge to developing standard measurement indicators that could be used to understand the magnitude, determinants and outcome of women’s empowerment at the national or global level.^11,12^ Nonetheless, a recent international report indicated that only 60.7% of women globally are empowered to achieve their full potential.^12^ The equivalent figure for SSA is 49.8%, compared with 76.3% for Europe and North America. The report covered 114 countries using 10 indicators from five women’s empowerment dimensions.^12^ Women’s empowerment is one items of the 2030 Sustainable Development Goal 5, achieve gender equality and empower all women and girls.^10^

Research on the relationship between women’s empowerment and care seeking for childhood health conditions are limited and show mixed findings. A study done using the Institute for Health Metrics and Evaluation's database of 161 countries indicated that women's political empowerment has a relevant effect on immunization for low-income and least-developed countries.^13^ A study in Zimbabwe, however, found no significant association between women’s empowerment and childhood vaccinations and treatment for diarrhoea.^11^

However, several other studies have assessed the effect of women’s empowerment on the nutritional status of children. A systematic review of studies in SSA^14^ and another comparative study in the region^15^ indicated women’s empowerment has a significant positive effect on childhood anthropometric indicators. A study in Gambia showed women who reported accepting wife-beating had 69% and 66% greater odds of having stunted and underweight children, respectively.^16^ In Peru, a study indicated that a low level of women's autonomy was significantly associated with a higher probability of anaemia in children <5 y of age.^17^ Studies conducted in Ethiopia also indicated that women’s empowerment had a significant protective effect on childhood malnutrition.^18,19^

Although Ethiopia has decreased mortality in children <5 y of age by >75% since 1990,^1^ it is still categorized among the global high-burden states.^1,20^ It is one of five countries where half of all deaths in children <5 y of age occurred in 2018.^1^ In 2021 alone, 178 000 children <5 y of age died in the country.^1^

Yet the prevalence of healthcare utilization for illnesses in children <5 y of age remains low in Ethiopia.^5,20^ The 2016 Ethiopia Demographic and Health Survey (EDHS) report indicated that among children <5 y of age in the country, 7% had ARI symptoms, 14% had fever and 12% had diarrhoea in the 2 weeks before the survey.^20^ According to the report, treatment was sought for 31%, 35% and 44% of children with ARI symptoms, fever and diarrhoea, respectively. The country also has a lower level of women’s empowerment. The 2016 EDHS report indicated that only 37% of all women and 70.6% of currently married women were fully empowered with attitudes towards wife-beating and participation in household decision-making, respectively.^20^

Ethiopia has adopted several child survival strategies, including the integrated management of newborn and childhood illnesses approach. The Health Sector Transformation Plan, the National Newborn and Child Development Strategy 2021–2025 and the implementation of a national action plan that targeted pneumonia and diarrhoea, the two major contributors (25%) of mortality in children <5 y of age in the country, are some of the national programs.^20,21^ Nevertheless, Ethiopia is known for its high mortality rate in children <5 y of age, low level of healthcare seeking for childhood illnesses and low prevalence of women’s empowerment.

Most studies on the relationship between women’s empowerment and child health have employed childhood anthropometric indicators as the outcome variable. This study aims to assess the association of women’s empowerment with healthcare seeking for diarrhoea, fever and ARI symptoms, the three major causes of mortality in children <5 y of age. To our knowledge, no previous study has examined the relationship between women’s empowerment and healthcare seeking for illnesses in in children <5 y of age.

## Methods

### Source of data

This study employed a retrospective cross-sectional survey data analysis using the Kids Recode (KR) dataset from the 2016 EDHS.^20^ All women 15–49 y of age were eligible to be interviewed. The sampling frame for the survey was the 2007 Population and Housing Census (PHC) frame, which contains a complete list of all census enumeration areas (EAs). The sample was stratified (urban and rural) and selected in two stages. At the first stage of sampling, a total of 645 (202 urban, 443 rural) EAs were selected using the probability proportional to size selection technique. A fixed number of 28 households per cluster was chosen in the second stage.

Data collection took place from 18 January 2016 to 27 June 2016. It was done with a face-to-face interview using a computer-assisted personal interviewing data collection system. A total of 15 683 women were interviewed, with a 95% response rate. Information on the health status of children was collected from these women. The survey used specially constructed weights for the sample selection. The women’s individual sample weight was used for this study.^20^ Ethiopia conducted a mini-DHS in 2019. However, the survey did not collect data on women’s empowerment and childhood illnesses.

### Measurements and variables

#### Outcome variable

The outcome variable for this study was utilization of healthcare services for the illnesses of diarrhoea, fever and ARI symptoms in children <5 y of age that occurred in the 2 weeks before the 2016 EDHS. Initially, health seeking for each illness was recoded as ‘yes’ (value 1) if women sought healthcare and ‘no’ (value 0) otherwise. The results were then merged to produce the total number of illnesses in children <5 y of age for which women sought healthcare. The values range from 0–3 (0, women did not seek healthcare for any illness; 3, women sought healthcare for all illnesses).

#### Predictor variables

The main predictor variable for this study was women’s empowerment. Based on a literature review and relevance to Ethiopia, 21 variables were initially identified as women’s empowerment indicators. Then, data screening and transformation was done to test for the adequacy of each selected variable. Five variables—respondent participates in decisions on spending husband’s earnings, participates in decisions on spending her own earnings, is employed in a regular paid job, is covered by health insurance and whether the husband helps with household chores—were excluded from this study mainly due to the small sample size and associated high missing values. Accordingly, the total number of women’s empowerment indicator variables was reduced to 16.

Following the data management, principal component analysis (PCA) was done using oblimin rotation to identify the components and the variables that aggregate the evidence within each women’s empowerment domain. Correlation analysis was done to test if the variables were fit to be used for PCA. The PCA (supplemented by parallel analysis) clustered the variables into four empowerment components: decision-making, attitude towards wife-beating, information education and communication (IEC) and durable asset ownership (Table 1).

**Table 1.** Domain-based women’s empowerment indicator variables and definitions.

Domain	Indicator	Definition of empowered
Decision making (3 items)	Decision on large household purchase	The woman participates in decisions on large household purchases alone or jointly with her partner
	Decision on own healthcare	The woman participates in decisions on her own healthcare alone or jointly with her partner
	Decision on visit to family/relatives	The woman participates in decisions on visits to her family or relatives alone or jointly with her partner
Attitude towards wife-beating (5 items)	Woman argues with her husband	The woman thinks that wife-beating is not justified if the woman argues with her husband
	Woman neglects the children	The woman thinks that wife-beating is not justified if the woman neglects the children
	Woman goes out without telling her husband	The woman thinks that wife-beating is not justified if the woman goes out without telling her husband
	Woman burns food	The woman thinks that wife-beating is not justified if the woman burns food
	Woman refuses to have sex with her husband	The woman thinks that wife-beating is not justified if the woman refuses to have sex with her husband
IEC indicators (6 items)	Newspaper	Reads the newspaper sometimes
	Television	Watches television sometimes
	Education level	Education level secondary and above
	Radio	Listens to radio sometimes
	Bank account	Has a bank account
	Cell phone	Has a cell phone
Durable asset ownership (2 items)	House	Owns house alone or jointly with her partner
	Land	Owns land alone or jointly with her partner

Items in each domain were then merged and transformed into dichotomous yes/no variables to produce domain-based women’s empowerment indicators. For this study, a woman is considered empowered if she is empowered with all items in each domain (fully empowered). For example, merger of the three ‘participation in decision-making’ women’s empowerment indicator items produces a scale ranging from 0 to 3. Values of 0–2 were recoded as 0 (not empowered) and a value of 3 was recoded as 1 (empowered).

#### Covariates

Based on the literature review, maternal and child sociodemographic variables (age of the child, sex of the child, residence, household wealth), maternal obstetric factors (number of children living in the house, desire for more children) and the socioeconomic factor (access to healthcare is not a big problem) were included as covariate variables. Whether ‘access to healthcare’ is not a problem was converted into a dichotomous variable by amalgamating problems related to distance, money, permission and women not wanting to go alone. It was then assigned ‘yes’ (value 1) if the respondent had no problem and ‘no’ (value 0) if she had at least one such problem. Selection of the covariates was determined by availability of the variable in the dataset.

### Statistical analysis

Following the PCA, a complex sample analysis plan was made specifying the sample weight for individual women, sample strata for sampling errors and the clusters variable based on the EDHS sample design information.^20,22^ Complex sample frequency, descriptive and crosstab were done to describe the sample by background characteristics and healthcare seeking for childhood illnesses. The bivariate χ^2^ test was used to assess for the presence of statistically significant (p<0.05) differences in health service utilization for childhood illnesses.

Univariate and multivariate complex samples ordinal regression analysis (CSORDINAL) was done to measure the predictors effect size on the outcome variable, with the corresponding cumulative odds ratio (cuOR) and 95% confidence intervals (CIs). The Taylor series linearization method was used for variance estimation. No observation deletion or suppression was made in the analysis.^23^ Data analysis was done using Statistical Software for Social Sciences version 27 (IBM, Armonk, NY, USA).

The EDHS collected data from women currently married or living with a man for its assessment of the women’s empowerment indicator ‘participation in household decision-making’. Accordingly, our study was limited to this subpopulation of respondents.

## Results

### Descriptive analysis

**Figure 1.**
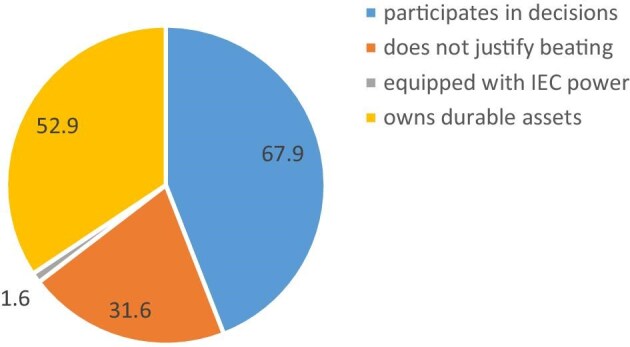
Percentage distribution of children <5 y of age by maternal empowerment status.

There were 9894 (unweighted) children <5 y of age living with mothers currently married or in union. The majority (67.9%) of the children had mothers fully empowered with household decision-making, while only 1.6% were from women fully empowered with the IEC domain of empowerment (Figure 1).

Among these children, 11.7% (n=1157) had diarrhoea, 14.0% (n=1390) had fever and 6.6% (n=656) had ARI symptoms in the 2 weeks before the EDHS. Healthcare seeking was seen in 44.5% of children with diarrhoea, 35.3% of children with fever and 29.6% of children with ARI symptoms (Table 2).

**Table 2.** Description of the study sample by healthcare utilization for illnesses in children <5 y of age.

	Children received care by type of illness, %		
Descriptive features	Diarrhoea (n=1157)	Fever (n=1390)	ARI (n=656)
Healthcare seeking	44.5	35.3	29.6
Healthcare sought on the same or next day		14.9	
ORS given	29.1		
Zinc given	32.8		
Antibiotic given		28.5	

No data for blank spaces.

Merging maternal healthcare seeking for each illness (diarrhoea, fever and ARI) returned 2268 (unweighted) cases; because it is the mother who sought healthcare for these children, the number (2268) also represents mother–child pairs of cases. While 61.9% of the cases received no healthcare, 2.9% received healthcare for three childhood illnesses (Table 3). Among these children, 66.7% (n=1513) had one childhood illness, while 7.9% (n=179) experienced all three illnesses in the 2 weeks before the survey. As the number of illnesses encountered increased, maternal utilization of healthcare for at least one childhood illness increased as well (Table 3).

**Table 3.** Description of the sample by number of illnesses encountered and number who sought healthcare.

	Illnesses for which women sought healthcare, %				
Illnesses encountered, n	0	1	2	3	Total cases, N
1	68.5	31.5			1513
2	52.8	13.5	33.7		576
3	35.2	21.3	6.8	36.7	179
Total	61.9	26.1	9.1	2.9	2268

Among the 2268 children, 51.9% were male and 89.3% were rural residents. The magnitude of women’s empowerment, stratified by healthcare seeking for illness in children <5 y of age, showed a slight change compared with the magnitude from the complex sample descriptive frequency. In the χ^2^ test, women’s empowerment with attitude towards wife-beating and ownership of durable assets domains showed a significant relationship with healthcare seeking for illnesses in children <5 y of age (Table 4).

**Table 4.** Description of the study sample by background characteristics and the number of illnesses for which the respondent sought healthcare.

	Illnesses for which women sought healthcare, % (N=2268 unless indicated)				
Predictors	0	1	2	3	Total sample (%)
**Empowerment indicators**					
Participation in household decision	(χ^2^=1.12, p=0.91)				
No	63.1	24.4	9.5	2.9	36.0
Yes	61.6	26.6	9.0	2.9	64.0
Does not justify wife-beating	**(χ^2^=39.37, p=0.00)**				
No	66.3	24.1	7.4	2.2	68.3
Yes	52.4	30.5	12.8	4.4	31.7
Equipped with IEC power	(χ^2^=2.17, p=0.57)				
No	62.3	25.8	9.1	2.9	98.6
Yes	53.3	26.9	16.8	3.0	1.4
Respondent owns durable assets	**(χ^2^=20.78, p=0.01)**				
No	57.5	30.1	9.1	3.3	48.7
Yes	66.5	21.7	9.2	2.6	51.3
**Sociodemographic indicators**					
Age of child (months)	(χ^2^=20.68, p=0.11)				
0–11	57.1	29.4	10.0	3.5	24.7
12–35	60.1	26.7	10.3	2.9	44.7
36–59	69.2	21.5	7.0	2.4	30.6
Sex of child	(χ^2^=0.72, p=0.94)				
Male	61.3	26.5	9.4	2.9	51.9
Female	63.0	25.0	9.0	2.9	48.1
Residence	**(χ^2^=50.54, p<0.00)**				
Rural	64.8	23.8	8.5	2.9	89.3
Urban	40.1	42.2	14.7	3.1	10.7
Household wealth status	**(χ^2^=53.49, p=0.00)**				
Lower	67.7	22.8	7.2	2.2	43.0
Middle	66.5	21.8	7.0	4.8	21.4
Higher	52.7	31.7	12.9	2.7	35.6
Healthcare access not a problem	**(χ^2^=54.54, p<0.00)**				
No	66.1	22.7	8.0	3.2	77.4
Yes	48.4	36.4	13.3	1.9	22.6
Children <5 y of age in the house, n (N=2229)	**(χ^2^=24.60, p=0.04)**				
1	55.9	30.4	10.2	3.5	42.3
2	66.6	22.3	8.7	2.4	42.8
3–6	67.2	22.3	8.1	2.3	14.9
Desire for more children (n=2139)	(χ^2^=4.78, p=0.50)				
No	64.7	23.9	8.8	2.6	38.0
Yes	59.7	27.3	10.0	3.1	62.0

Significant values in bold.

### CSORDINAL univariate regression

Children whose mothers were empowered with attitude towards wife-beating had an increased probability of receiving healthcare for all childhood illnesses in the univariate regression (β=0.60, cuOR 1.82, p<0.01). Maternal ownership of durable assets, however, was found to have opposite effect (β=−0.34, cuOR 0.71, p=0.01) (Table 5).

**Table 5.** CSORDINAL univariate regression analysis results.

		CSORDINAL univariate regression		
Predictors	β	cuOR	95% CI	TPL p-value
**Empowerment indicators**				
Participation in household decision				
No	0.07	1		0.95
Yes		1.08	0.82 to 1.41	
Does not justify wife-beating				
No	**0.60**	**1**		0.92
Yes		**1.82**	1.40 to 2.40	
Equipped with IEC power				
No	0.44	1		0.61
Yes		1.55	0.55 to 4.33	
Respondent owns durable assets				
No	**−0.34**	1		0.31
Yes		**0.71**	0.56 to 0.91	
**Sociodemographic indicators**				
Age of child (months)				
0–11		**1**		0.99
12–35	**−0.48**	**0.62**	0.44 to 0.87	
36–59	−0.12	0.89	0.66 to 1.21	
Sex of child				
Male	−0.06	1		0.95
Female		0.94	0.73 to 1.21	
Residence				
Rural	**0.91**	**1**		0.05
Urban		**2.50**	1.81 to 3.44	
Household wealth status (lower, middle, higher)	**0.32**	**1.37**	1.18 to 1.60	0.41
Healthcare access not a problem				
No	**0.66**	**1**		0.03
Yes		**1.93**	1.47 to 2.55	
Children <5 y of age in the house (1, 2, 3–6)	**−0.29**	**0.75**	0.62 to 0.91	0.68
Desire for more children				
No	0.21	1		0.95
Yes		1.23	0.92 to 1.65	

TPL: test of parallel lines.

Numbers rounded to the nearest two decimals.

Significant values in bold.

### CSORDINAL multivariate regression

A total of 2101 children were included in the CSORDINAL multivariate model, with a 7.4% missingness rate. Children whose mothers were empowered with an attitude towards wife-beating were found to have a 59% greater probability of receiving healthcare services for all the illnesses in children <5 y of age (β=0.46, cuOR 1.59, p<0.01). Children of mothers with no healthcare access problems were also more likely to receive health services for all illnesses in children <5 y of age (β=0.42, cuOR 1.52, p=0.01) (Table 6).

**Table 6.** CSORDINAL multivariate regression analysis results.

		CSORDINAL multivariate regression	
Predictors	β	cu-OR	95% CI
**Empowerment indicators**			
Participation in household decisions			
No	−0.05	1	
Yes		0.95	0.72 to 1.23
Does not justify wife-beating			
No	**0.46**	**1**	
Yes		**1.59**	1.20 to 2.10
Equipped with IEC power			
No	−0.48	1	
Yes		0.62	0.21 to 1.85
Respondent owns durable assets			
No	−0.26	1	
Yes		0.77	0.59 to 1.02
**Sociodemographic indicators**			
Age of child (months)			
0–11		**1**	
12–35	**−0.39**	**0.67**	0.47 to 0.97
36–59	−0.06	0.94	0.68 to 1.31
Sex of child			
Male	−0.11	1	
Female		0.90	0.69 to 1.17
Residence			
Rural	0.38	1	
Urban		1.46	0.91 to 2.35
Household wealth (lower, middle, higher)	0.12	1.13	0.91 to 1.37
Healthcare access not a problem			
No	**0.42**	**1**	
Yes		**1.52**	1.10 to 2.09
Children <5 y of age in the house (1, 2, 3–6)	−0.16	0.85	0.71 to 1.03
Desire for more children			
No	0.11	1	
Yes		1.12	0.83 to 1.49

p-Value for test of parallel lines=0.07.

Numbers are rounded to nearest two decimal places.

Significant values in bold.

## DISCUSSION

This study assessed the relationship between women’s empowerment and healthcare seeking for diarrhoea, fever and ARI symptoms, the three common causes of childhood mortality. Our findings indicated that the prevalence of care seeking for childhood illnesses ranged from 30% to 45%. Less than 33% of the children received ORS, zinc or antibiotics for their illnesses. The prevalence of healthcare seeking in our study is lower than in the UNICEF report for SSA (46–60%)^5^ and the ecological study report for the region (49%).^4^ However, our findings are better than similar research from Nigeria and Kenya.^6,7^ The rationale for the variation in findings could be due to the difference in health awareness, socio-economic status and women's empowerment status.

The results from the CSORDINAL multivariate analysis showed that women’s empowerment with attitude towards wife-beating has a positive effect on healthcare utilization towards all diseases in children <5 y of age. This is in line with other studies^14–19^ that indicated women’s empowerment has a protective effect on childhood malnutrition. However, the outcome variable for our study was health seeking for illnesses in children <5 y of age. hence caution should be exercised when comparing.

The women’s empowerment indicators—participation in household decision-making, ownership of durable assets and IEC power—were found to have no significant association with health seeking for illnesses in children <5 y of age in the final multivariate model. This finding is similar to that of a study from Zimbabwe,^11^ which indicated that women’s empowerment with decision-making or maternal ownership of durable assets had no significant association with both childhood vaccination uptake and treatment for diarrhoea.

However, the research findings above deviate from the underlying theoretical foundation of women’s empowerment.^8–10^ The absence of standard measurement indicators of empowerment may contribute to the deviation of the research findings in general. Another possible explanation for the finding related to the empowerment indicator ‘participation in household decision-making’ may lie in the definition of the indicator itself. A woman is said to be empowered if she makes decisions alone or jointly with her partner. Joint decision-making could be considered communicative, participatory and equitable. However, the degree of women’s involvement that qualifies a ‘joint decision-making’ cannot be quantified objectively. This could introduce research bias. Accordingly, we suggest that although some household issues require couple's joint decision-making, independent decision-making by women alone should be considered as a women’s empowerment indicator. Social desirability bias, where the respondent answers as if she were participating in household decision-making, but in reality she did not, might be another source of bias for the findings.

Some authors classify women’s empowerment variables as empowerment indicators and empowerment enablers: participation in household decision-making and attitude towards wife-beating are usually considered as empowerment indicators by researchers.^11,12,24^ Our finding that maternal ownership of durable assets and IEC power had no significant association with care seeking for illnesses in children <5 y of age could indicate that owning empowerment enablers does not directly translate to women’s empowerment. Especially in Africa, that a woman in a union owns durable assets does not necessarily mean that she is able to give away, rent out, sell or buy more assets alone or jointly. Less than 2% of the respondents in this study were fully empowered in the IEC domain of women’s empowerment. This low level of women’s empowerment status could affect the power of the test as well.

Our study found that a unit increase in age of children ages 12–35 months significantly decreases the probability of healthcare seeking towards all illnesses in children <5 y of age compared with children ages 0–11 months. However, a similar decreasing trend was observed for children ages 36–59 months compared with those ages 0–11 months. This may indicate that mothers are more sensitive to the illnesses of younger children compared with the older age groups.

As the number of children <5 y of age living in the household increases, healthcare seeking was found to decrease significantly. This may be due to higher socio-economic demands related to providing care for many children in the house. The study also showed that respondents with no healthcare access problem were more likely to seek care towards all illnesses in children <5 y of age.

### Strengths and limitations of the study

This study tried to address the research gap on the relationship between women’s empowerment and healthcare seeking for diarrhoea, ARI symptoms and fever, the three major causes of mortality in children <5 y of age. This makes our research the first of its kind. We extracted data from the EDHS 2016 Kids Recode dataset for this study. The findings can therefore be inferred as the health-seeking behaviour of mothers for illnesses in children <5 y of age in Ethiopia. Lack of standard women’s empowerment indicators is a limitation of this study. We also cannot establish a cause-and-effect relationship from a cross-sectional research design.

## Conclusions

This study revealed that women’s empowerment has a significant association with maternal healthcare seeking for illnesses in children <5 y of age. The findings indicate that a lack of empowerment not only affects the life of the victim, it is also significant to others in the household, including children. It implies that empowering women is empowering the family. Hence, for women to make autonomous strategic life decisions, including healthcare seeking for children, significant attention should be given to women’s empowerment. We believe that understanding and addressing deterrents to maternal healthcare seeking behaviour could contribute significantly to the prevention and control of childhood mortality in SSA.

## Data Availability

The dataset used for this study is available from https://dhsprogram.com. Accessing the dataset requires registration and submission of research protocols. Further information about the data can be found at https://dhsprogram.com/data/Access-Instructions.cfm.
